# A fast *in situ* hybridization chain reaction method in *Drosophila* embryos and ovaries

**DOI:** 10.1080/19336934.2024.2428499

**Published:** 2024-12-05

**Authors:** Kyohei Mikami, Yasuhiro Kozono, Masaki Masukawa, Satoru Kobayashi

**Affiliations:** a Degree Programs in Life and Earth Sciences, Graduate School of Science and Technology, University of Tsukuba, Tsukuba, Ibaraki, Japan; b Life Science Center for Survival Dynamics, Tsukuba Advanced Research Alliance (TARA), University of Tsukuba, Tsukuba, Ibaraki, Japan

**Keywords:** Drosophila melanogaster, *in situ* HCR, *in situ* hybridization chain reaction, embryo, ovary, rapid staining

## Abstract

The *in situ* hybridization chain reaction (isHCR) is a powerful method for visualizing mRNA in many species. We present a rapid isHCR method for *Drosophila* embryos and ovaries. Ethylene carbonate was added to the hybridization buffer to facilitate the hybridization reaction, and a modified short hairpin DNA was used in the amplification reaction; these modifications decreased the RNA staining time from 3 days to 1 day. This method is compatible with immunohistochemistry and can detect multiple mRNAs. The proposed method could significantly reduce staining time for *Drosophila* researchers using isHCR.

## Introduction

The *in situ* hybridization chain reaction (isHCR) is a powerful method for visualizing mRNA; its advantages include multiplex capability, high contrast, subcellular resolution, quantitative analysis capabilities, and compatibility with various samples [1–4]. The isHCR is mediated by two types of hairpin DNA (H1 and H2) labelled with fluorescent molecules and DNA probes that hybridize to the target mRNA (Supplemental Figure S1). The hairpin DNA consists of toehold, stem, and loop domains, and the DNA probe contains a sequence complementary to the target mRNA and an initiator sequence that binds to H1. Binding of the toehold and stem domain of H1 to the initiator sequence of the DNA probe opens the H1 hairpin, which triggers the polymerization of H1 and H2 through a hybridization chain reaction that results in the formation of a fluorescent polymer at the location of the target mRNA [3–6]. Third generation isHCR (3rd-gen isHCR), which uses split binding and initiator probes to decrease the background signal, is currently widely used [4]. In *Drosophila* embryos and ovaries, DNA probe hybridization and hairpin DNA amplification are performed overnight, and the experiment is completed in 3 days [1,4,7,8]. The applicability of isHCR is thus limited by the long staining time, although it remains a powerful tool.

The recently developed *in situ* short hairpin HCR (is-shHCR) is a modification of the 3rd-gen isHCR. In is-shHCR, H1 and H2 are shortened from 72 nt to 42 nt, thereby reducing the cost of hairpin DNA synthesis [3]. The is-shHCR is used as the standard protocol with an amplification time of 2 hours in sections of mouse, eel, zebrafish brain, mouse intestinal epithelial cells and chum salmon cells [9–14]. However, whether is-shHCR can be used in whole-mount samples, such as *Drosophila* embryos and ovaries, remains unclear.

Here, we report a new isHCR method for *Drosophila* whole embryos and ovaries. Instead of toxic formamide, ethylene carbonate (EC), which is a non-toxic solvent and facilitates the hybridization reaction [15], was included in the is-shHCR protocol to decrease the reaction time. This method, termed EC-isHCR, reduces RNA staining time from 3 days to 1 day. In addition, we eliminated treatment with xylene and proteinase K, as well as the post-fixation step, which are used for permeabilization in conventional isHCR [1,4,16]. The elimination of these treatments resulted in the detection of strong RNA signals. EC-isHCR was compatible with immunohistochemistry, and several mRNAs were detected in embryos at different developmental stages and in ovaries, suggesting the robustness of the method.

## Materials and methods

### Fly stocks

Flies were maintained on standard *Drosophila* medium at 25°C. The fly strain used in this study was *y w*.

### Split-initiator DNA probes

The split-initiator DNA probes were designed as described previously [3,12]. Probes were designed semi-automatically using R. Details of this pipeline are shown on Github (https://github.com/Dro-g/EC-isHCR_ProbeDesign). Briefly, sequences complementary to the target mRNAs were obtained, their off-target potential was evaluated using Blastn, and initiator sequences were added. The S45 initiator sequence (5*'*-CCTCCACGTTCCATCTAAGCT-3*'*) corresponding to SaraFluor™ 488-conjugated S45 hairpin DNA (Nepagene, Cat. No. IPL-G-S45) was used in probes for *P-element induced wimpy testis* (*piwi*), *nanos* (*nos*), *Ultrabithorax* (*Ubx*), and *enhanced green fluorescent protein* (*EGFP*) mRNA, and the A161 initiator sequence (5'-GGTACGCGAAGGTAGGTGTAA-3') corresponding to ATTO647N-conjugated A161 hairpin DNA (Nepagene, Cat. No. IPL-B-A161) was used in probes for *germ cell less* (*gcl*), *engrailed* (*en*), and *K10* mRNA (Supplemental Table S1). Five sets of split-initiator probes were designed for *EGFP* mRNA, 10 sets for *nos*, *gcl*, *Ubx*, *en*, and *K10* mRNA, and 20 sets for *piwi* and *ovo* mRNA. For mRNAs with weak signals, the number of probe sets was increased to enhance the signal. The probes were synthesized as DNA oligos, purified by Oligonucleotide Purification Cartridge (OPC) (Eurofins genomics), and stored at −20°C.

### Fixation of embryos and ovaries

Embryos were collected and dechorionated in a sodium hypochlorite solution. The dechorionated embryos were fixed in 1:1 heptane:fixative [4% paraformaldehyde in PBS (137 mM NaCl, 2.7 mM KCl, 10 mM Na_2_HPO_4_^ · ^12H_2_O and 1.8 mM KH_2_PO_4_)] for 30 minutes. Vitelline membranes were removed by vigorously shaking the embryos in 1:1 methanol:heptane. The embryos were then rinsed with methanol three times and stored in methanol at −20°C.

Ovaries were dissected from adult flies and treated with fixative for 20 minutes. The fixed ovaries were rinsed with PBSTr (PBS containing 0.1% Triton X-100) three times, followed by methanol:PBSTr at ratios of 1:3, 1:1, and 3:1 and storage in methanol at −20°C at least overnight (up to 1 week).

### EC-isHCR and immunohistochemistry

The fixed embryos and ovaries were sequentially rinsed with 3:1, 1:1, and 1:3 methanol:PBSTr. The samples were rinsed twice with PBSTr, then with 1:1 PBSTr:SSCT [0.1% Tween-20 in 1× SSC (150 mM NaCl and 15 mM Trisodium Citrate Dihydrate, pH 7.0)], and finally with SSCT [rinse in PBSTr could be omitted (Supplementary Figure S2)].

For the experiments with xylene, proteinase K, and fixative treatment [1,4], the fixed embryos were rinsed four times with ethanol, followed by rinsing with 3:2 xylene:ethanol and incubation in 3:2 xylene:ethanol for 1 hour. The embryos were then rinsed with ethanol and washed three times with ethanol for 5 minutes each. After rinsing with methanol and washing three times with methanol for 5 minutes each, the embryos were rinsed with 1:1 methanol:PBSTw (PBS containing 0.1% Tween-20) and washed once with PBSTw for 10 minutes followed washing twice with PBSTw for 5 minutes. They were then treated with proteinase K solution (PBSTw containing 4 μg/ml proteinase K) for 7 minutes and post-fixed with the fixative for 25 minutes. The post-fixed samples were rinsed with PBSTw, washed five times with PBSTw for 5 minutes, and rinsed with PBSTw, 1:1 PBSTw:SSCT and SSCT, as described above.

The samples treated as described above were pre-hybridized in pre-warmed hybridization buffer [15% ethylene carbonate (Sigma-Aldrich, Cat. No. E26258), 5× SSC, 0.1% Tween-20, 50 μg/ml heparin (Sigma-Aldrich, Cat. No. H3393-25KU), 1× Denhardt’s solution (Fujifilm-Wako, Cat. No. 043-21871)] for 30 minutes at 45°C. Then, the samples were hybridized in pre-warmed probe solution for 2 hours at 45°C (for ‘3-days staining’ in ‘Image contrast of EC-isHCR’ section, the samples were hybridized for 16 hours at 45°C). To make a hybridization solution, each probe set (for *piwi*, *nos*, *Ubx, gcl*, *en*, and *K10* mRNA) was diluted in hybridization buffer at 20 nM. Each probe set for *EGFP* mRNA was diluted in hybridization buffer at 80 nM. To standardize the total amount of probes between the experimental and control groups, we adjusted the dilution rates of *piwi* and *EGFP* probe set according to the number of each probe sets. The hybridization solutions were warmed at 95°C and immediately cooled on ice water [the probe denature step could be omitted (Supplementary Figure S2)]. After hybridization, the samples were washed twice with hybridization buffer for 10 minutes each at 45°C, followed by rinsing with 1:1 hybridization buffer:SSCT and twice with SSCT at room temperature. Then, the samples were washed with SSCT for 10 minutes at room temperature. For the amplification reaction, SaraFluor™488-conjugated S45 or ATTO647N-conjugated A161 hairpin DNA pairs (H1 and H2) were used. The hairpin DNA pairs were separately heated to 95°C for 2 minutes, and gradually cooled to 65°C for 15 minutes and to 25°C for 40 minutes before use (thermal cycler setting: cooling the hairpin DNA to 95°C for 2 minutes, then −2°C/minute to 65°C, then −1°C/minute to 25°C and maintain at 25°C). The samples were incubated with amplification buffer (Nepagene, Cat. No. IPL-AB) containing H1 and H2 hairpin DNAs (1/50 volume of amplification buffer) for 1.5–2 hours at 25°C (for ‘3-days staining’ in ‘Image contrast of EC-isHCR’ section, the samples were incubated for 12 hours at 25°C). For multiplex staining, SaraFluor™488-conjugated S45 and ATTO647N-conjugated A161 hairpin DNA pairs were added simultaneously. After amplification, the samples were washed three times with SSCT for 10 minutes each at room temperature and mounted in VECTASHIELD Antifade Mounting Medium (Vector Laboratories, Cat. No. H-1000).

For immunohistochemistry, after amplification, the samples were rinsed with SSCT and 1:1 SSCT:PBSTr, followed by three washes with PBSTr for 15 minutes each at room temperature. The samples were then incubated with blocking solution (PBS containing 2% BSA, 0.1% Tween-20, and 0.1% Triton X-100) for 1 hour. After blocking, the samples were incubated overnight with blocking solution containing an anti-Vasa chicken antibody [17] (1:1000) at 4°C. The samples were rinsed three times with PBSTr, washed three times for 15 minutes each with PBSTr, and incubated overnight with blocking solution containing Alexa Fluor 633-conjugated goat anti-chicken (1:500; Thermo Fisher Scientific, Cat. No. A21103) at 4°C. The samples were rinsed three times with PBSTr, washed three times for 15 minutes each with PBSTr, and mounted in VECTASHIELD Antifade Mounting Medium.

A detailed description of the methods is provided in the supplementary text.

All images were obtained using a TCS-SP8 confocal microscope (Leica Microsystems). Images were processed with Fiji software [18].

### Re-analysis of RNA-sequencing data

We re-analysed RNA-sequencing (RNA-seq) data from primordial germ cells (PGCs) of stage 4–5 of *EGFP-vas* transgenic line [19,20]. These data have been deposited in DDBJ Bio Project database under Accession No. DRA009066. Raw reads were processed using Trimmomatic, and then were pseudo-aligned to the *Drosophila* transcriptome (Flybase; dmel-all-transcript-r6.17.fasta) by using Kallisto (ver. 0.43.1) with default settings [21]. Gene-level expression values were summarized using the summarizeToGene function of tximport [22,23], with the reference table of FBtr and FBgn (fbgn_fbtr_fbpp_fb_2017_04.tsv).

## Results

### Development of EC-isHCR

**Figure 1.**
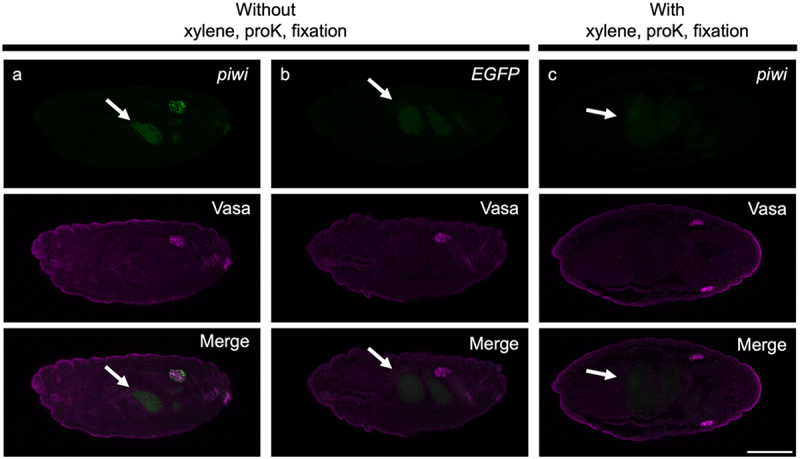
Visualization of *piwi* mRNA and Vasa protein by EC-isHCR and immunohistochemistry. EC-isHCR signals (green) and anti-Vasa protein signals (magenta) were detected in stage-16 embryos. Embryos were stained using probes for *piwi* mRNA (a), *EGFP* mRNA (b), and *piwi* mRNA with xylene, proteinase K (proK), and fixative treatments (c). Arrows indicate background signals in the midgut. Scale bar: 100 µm.

First, we stained for *piwi* mRNA in whole-mount embryos, in which *piwi* mRNA is confined to the embryonic gonads (BDGP: https://www.fruitfly.org). A *piwi* mRNA signal was observed within the gonads, marked by an antibody against the Vasa protein, a marker for primordial germ cells (PGCs) (Figure 1a). No signal was detected in the embryos with probe sets for *EGFP* mRNA, which is not endogenously expressed (Figure 1b). In embryos permeabilized with proteinase K and xylene and subjected to the post-fixation treatment, which are steps included in previous isHCR protocols [1,4], a *piwi* signal was not detected (Figure 1c). Thus, we omitted these treatments from the method. The results indicate that EC-isHCR is effective for visualizing *piwi* RNA in 1 day.

### Applicability of EC-isHCR

**Figure 2.**
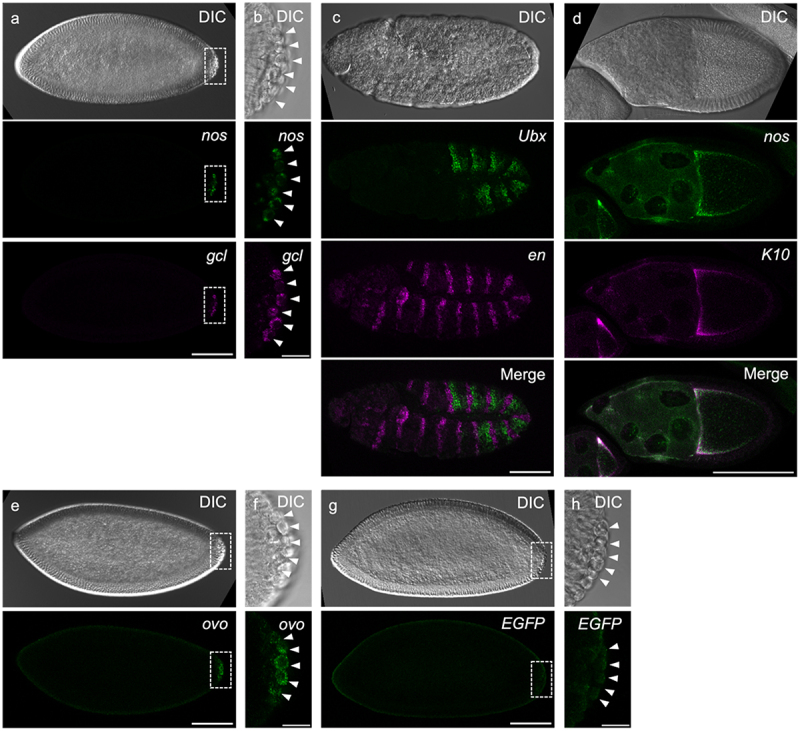
Detection of mRNAs using EC-isHCR in *Drosophila* whole-mount embryos and adult ovaries. (a–h) Differential interference contrast (DIC) images and ec-isHCR signals are shown. (a, b) *nos* (green) and *gcl* (magenta) mRNAs were detected in stage-5 embryo. (b) High magnification view of the region boxed in (a). (c) *Ubx* (green) and *en* (magenta) mRNAs were detected in stage-11 embryo. (d) *nos* (green) and *K10* (magenta) mRNAs were detected in stage-10 egg chamber of the adult ovary. (e-h) Stage-5 embryos were stained using probes for *ovo* mRNA (e, f), *EGFP* mRNA (g, h). (f, h) High magnification view of the region boxed in (e, g) respectively. Arrowheads indicate primordial germ cells. Scale bars: 100 µm (a, c, d, e, g) and 20 µm (b, f, h).

To test the robustness of EC-isHCR, we detected several mRNAs in whole-mount embryos at the blastoderm and germ band extension stages, as well as in whole-mount ovaries. In *Drosophila* blastoderm embryos, the mRNAs for *nos* and *gcl* were restricted to PGCs [24–26]. After germ band extension, two homeobox genes, *Ubx* and *en*, were expressed in stripes within different parasegments. *Ubx* mRNA is expressed in the anterior compartment of parasegments 6–12 [27], whereas *en* mRNA forms 15 segmental stripes in the posterior compartment of parasegments 1–15 [8,28,29]. The distribution of these mRNAs was visualized using EC-isHCR (Figure 2). In blastoderm embryos, *nos* and *gcl* mRNA signals were detected in the posterior pole and were restricted to PGCs (Figure 2a,b). The pattern of expression of *Ubx* and *en* mRNA observed in this study was consistent with that described previously [8,27–29] (Figure 2c). In stage 10 egg chambers from adult ovaries, *fs(1)K10* (*K10*) and *nos* localized to the anterior margin of oocytes, and *nos* was also expressed in nurse cells [24,30]. The EC-isHCR signals corresponding to *K10* and *nos* mRNA were located in regions previously reported to express *K10* and *nos* mRNA (Figure 2d). These observations demonstrate that EC-isHCR detected several mRNAs simultaneously in whole-mount embryos and ovaries.

We investigated whether EC-isHCR can detect mRNA which is functional but not highly expressed. Maternal *ovo* mRNA encodes a transcription factor and is necessary for PGC development [31]. When the RNA-seq data for normal PGCs at stage 4–5 [19] was re-analysed, we found that the expression level of maternal *ovo* mRNA was approximately 1.5% of *nos* mRNA and approximately 5% of *gcl* mRNA (Supplemental Table S2), indicating that maternal *ovo* mRNA was not highly expressed in PGCs. By using EC-isHCR, signals of *ovo* mRNA were detected in PGCs of stage-5 embryos (Figure 2e-h). This suggests that EC-isHCR has the sensitivity to detect mRNA which is not highly expressed.

### Image contrast of EC-isHCR

**Figure 3.**
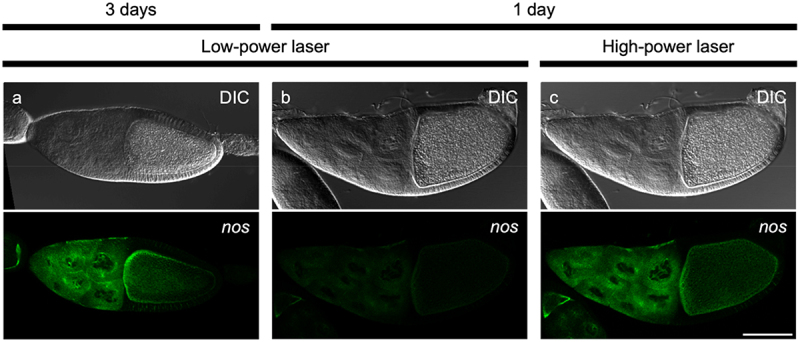
Visualization of *nos* mRNA using 3-day staining and EC-isHCR in whole-mount *Drosophila* adult ovaries. (a-c) Differential interference contrast (DIC) images and EC-isHCR signals are shown. *nos* mRNA (green) was detected in stage-10 egg chambers of adult ovaries. (a) The egg chamber was stained using 3-day staining method, and the image was captured with optimized confocal laser-microscopy settings (low-power laser). (b) The egg chamber was stained using EC-isHCR, and the image was taken under the low-power laser settings. (c) The egg chamber was stained using EC-isHCR, and the image was captured with optimized confocal laser-microscopy settings (high-power laser). Scale bars: 100 µm.

To assess contrast of images obtained by EC-isHCR, we compared EC-isHCR to a protocol in which both hybridization and amplification were performed overnight (3-days staining). Consistent with the previous observation that the long-term amplification produces intense signals [3], we found that the signal intensity obtained with 3-day staining was higher than that of our 1-day method (Figure 3a,b). However, there was no difference in image contrast under optimized conditions for confocal laser-microscopy (Figure 3c). These observations suggest that our method does not compromise the signal-to-noise ratio.

## Discussion

This study describes a fast method for whole-mount isHCR in *Drosophila* embryos and ovaries. Several detection methods in *Drosophila* embryos and ovaries have been reported previously, including isHCR, tyramide signal amplification (TSA) [32–34], and single-molecule FISH (smFISH), which uses multiple single-labelled short oligonucleotides [35–38]. The time from hybridization to mount is longer in conventional isHCR than in the other methods, with 3 days for isHCR, 2 days for TSA, and 1 day for smFISH [4,7,8,32,33,35]. We introduced modifications that reduced the staining time for isHCR, resulting in a faster isHCR method.

The proposed method is cost effective because we used a shorter fluorescently labelled hairpin DNA than that used for 3rd-gen isHCR [3,4,7,8]. Furthermore, the cost of the probe design can be reduced. We developed a semi-automated probe design pipeline for the first time (see Materials and methods, https://github.com/Dro-g/EC-isHCR_ProbeDesign). The probe design programme for is-shHCR used in previous studies is not publicly available and depends on the manufacturers.

The method designed in this study can be applied to various samples because it can detect mRNA in both embryos and ovaries without requiring sample-specific modifications. Furthermore, EC, which was used to shorten hybridization time, is not an insect-specific reagent and is used for hybridization in various species and cell types [15,39,40]. These features make the present method an important contribution towards establishing a fast isHCR method that is applicable to various species.

Several *in situ* hybridization methods have been developed, which enables the selection of the most appropriate method for each application. Here, we discuss advantages and disadvantages of TSA, smFISH, and isHCR. Both isHCR and TSA serve as effective methods for visualizing expression patterns of different mRNAs. isHCR and TSA are suitable for detecting various mRNAs, because the fluorescently labelled hairpin DNA and the tyramide reagent, which are used for isHCR and TSA, respectively, are common, regardless of the target mRNAs [7,25,26,38,41]. These methods can also detect low-copy mRNAs [3,42]. isHCR is superior to TSA regarding the experimental time ranges. isHCR uses oligo DNA probes, which can be synthesized rapidly, whereas TSA uses longer RNA probes, for which the templates require longer preparation times. In contrast, the applications differ between smFISH and isHCR. smFISH is unsuitable for the detection of various mRNAs because synthesizing fluorescently labelled short oligonucleotides for each target mRNA is costly. Furthermore, it is possible that smFISH is not effective for detecting low-copy mRNAs because the signals cannot be amplified. However, smFISH is useful for quantitative and subcellular resolution analyses [36,37]. Taken together, these findings suggest that isHCR is a cost- and time-saving method for many mRNA targets.

In summary, we provide a fast and cost-effective isHCR method for detection of multiple mRNAs in *Drosophila* embryos and ovaries. The proposed method is feasible for high-throughput staining, such as that used in gene expression screening and for validating the expression of candidate marker genes identified by single-cell RNA-seq. The present method could thus contribute to advancing a wide range of *Drosophila* studies.

## Supplementary Material

Supplemental Material

Supplementary_Figure_revise.docx

Supplementary_Table_revise.xlsx
