# Supplementary material for: A fast in situ hybridization chain reaction method in Drosophila embryos and ovaries
Source: Fly (Austin). 2024 Dec 5;19(1):2428499. doi: 10.1080/19336934.2024.2428499 (PMC11633216; doi:10.1080/19336934.2024.2428499)
Supplement: Supplemental Material [file KFLY_A_2428499_SM0882.docx]

**Materials and Methods (detailed version)**

A detailed description of the materials and methods, including information about procedures, notes, and equipment is provided. Procedures in which the temperature is not specified were performed at room temperature (approximately 23°C).

***Design of split-initiator DNA probes***

We developed a pipeline for designing probes semi-automatically using R (<https://github.com/Dro-g/EC-isHCR_ProbeDesign>).

Probes were synthesized as DNA oligos, purified by Oligonucleotide Purification Cartridge (OPC), and diluted in TE buffer (Eurofins Genomics). Probes were stored in aliquots of 100, 50, and 1 μM in TE buffer at −20°C.

***Equipment and setup***

- Equipment for standard immunohistochemistry in *Drosophila* embryos and ovaries
- Incubators (25°C and 45°C)

Setup: a rotator is placed in the 25°C incubator for amplification

- Thermal cycler

Setup: the thermal cycler should have a function for snap-cooling fluorescently labeled hairpin DNA. A program needs to be created to snap-cool the hairpin DNAs to 95°C for 2 minutes, followed by −2°C/minute to 65°C, then −1°C/minute to 25°C and maintain at 25°C.

***Fixation of embryos and ovaries***

1. Embryos
2. Collect embryos on grape juice/agar plates, place on a mesh, and dechorionate embryos in a sodium hypochlorite solution for 10–30 seconds.
3. Rinse embryos with MilliQ water.
4. Transfer embryos to 1:1 heptane:fixative in a 2-ml tube.
5. Incubate embryos with mixing for 30 minutes.
6. Remove the fixative, add 1 ml of methanol, and shake for 30–60 seconds to remove vitelline membranes.
7. Remove the liquid and rinse the embryos with methanol three times.
8. Store embryos in methanol at −20°C.
9. Ovaries
10. Dissect ovaries from adult flies.
11. Transfer ovaries to fixative in 2-ml tube.
12. Incubate ovaries with mixing for 20 minutes.
13. Rinse ovaries with PBSTr three times.
14. Rinse ovaries with 3:1, 1:1, and 1:3 PBSTr:methanol.
15. Store ovaries in methanol at −20°C.

***EC-isHCR***

Description of the procedure omitting the following steps: rinse with PBSTr before hybridization and denature the probe.

**Before hybridization**

1. Prewarm hybridization buffer at 45°C and amplification buffer at 25°C.
2. Rinse the fixed samples with 3:1, 1:1, and 1:3 methanol:SSCT.
3. Rinse samples once and wash with SSCT three times for 10 minutes each.
4. Prehybridize samples with 100 μl of hybridization buffer for 30 minutes at 45°C.

NOTE: remove SSCT as much as possible.

1. Prepare a hybridization solution and prewarm at 45°C.

**Hybridization**

1. Remove the hybridization buffer, add 100 μl of hybridization solution, and incubate for 2 hours.

NOTE: remove hybridization buffer as much as possible.

NOTE: During hybridization, snap-cool the fluorescently labeled hairpin DNA 1 (H1) and hairpin DNA 2 (H2), as described below (Procedure No.1 in Amplification Section).

1. Wash samples with hybridization buffer twice for 10 minutes at 45°C.
2. Rinse samples with 1:1 SSCT:hybridization buffer. Rinse samples with SSCT twice.
3. Wash samples for 10 minutes with SSCT.

**Amplification**

1. Snap-cool H1 and H2 (heated to 95°C for 2 minutes, and gradually cooled to 65°C for 15 minutes and to 25°C for 40 minutes).

NOTE: Incubate H1 and H2 separately.

1. Mix the amplification buffer with a vortex mixer for 3 seconds, three times.

NOTE: The amplification buffer should be mixed carefully due to its high viscosity.

1. Add H1 and H2 to the amplification buffer (1/50) to make an amplification solution. Mix the solution with a vortex mixer for 3 seconds three times.

NOTE: Prepare the amplification solution just before applying it to the sample.

1. Remove SSCT and add 100 μl of amplification solution.

NOTE: remove SSCT as much as possible.

1. Incubate for 2 hours at 25°C in the dark with rotation.

NOTE: The subsequent procedures should be performed under light protection.

**To mount**

1. Wash samples for 10 minutes with SSCT three times.
2. Mount samples with VECTASHIELD Antifade Mounting Medium (Vector Laboratories, Cat. No. H-1000) and store at 4°C.

***Immunohistochemistry***

For immunohistochemistry, the steps described below were performed after the amplification step.

1. Rinse samples with SSCT.
2. Rinse samples with 1:1 SSCT:PBSTr.
3. Wash samples with PBSTr three times for 15 minutes each.
4. Block samples with blocking solution for 1 hour.
5. Incubate samples with primary antibodies overnight at 4°C.
6. Rinse samples three times and wash with PBSTr three times for 15 minutes each.
7. Incubate samples with secondary antibodies overnight at 4°C.
8. Rinse samples three times and wash with PBSTr three times for 15 minutes each.
9. Mount samples with VECTASHIELD and store at 4°C.

**Buffers and solution**
