# Supplementary material for: A fast in situ hybridization chain reaction method in Drosophila embryos and ovaries
Source: Fly (Austin). 2024 Dec 5;19(1):2428499. doi: 10.1080/19336934.2024.2428499 (PMC11633216; doi:10.1080/19336934.2024.2428499)
Supplement: Supplementary_Figure_revise.docx [file KFLY_A_2428499_SM3937.docx]

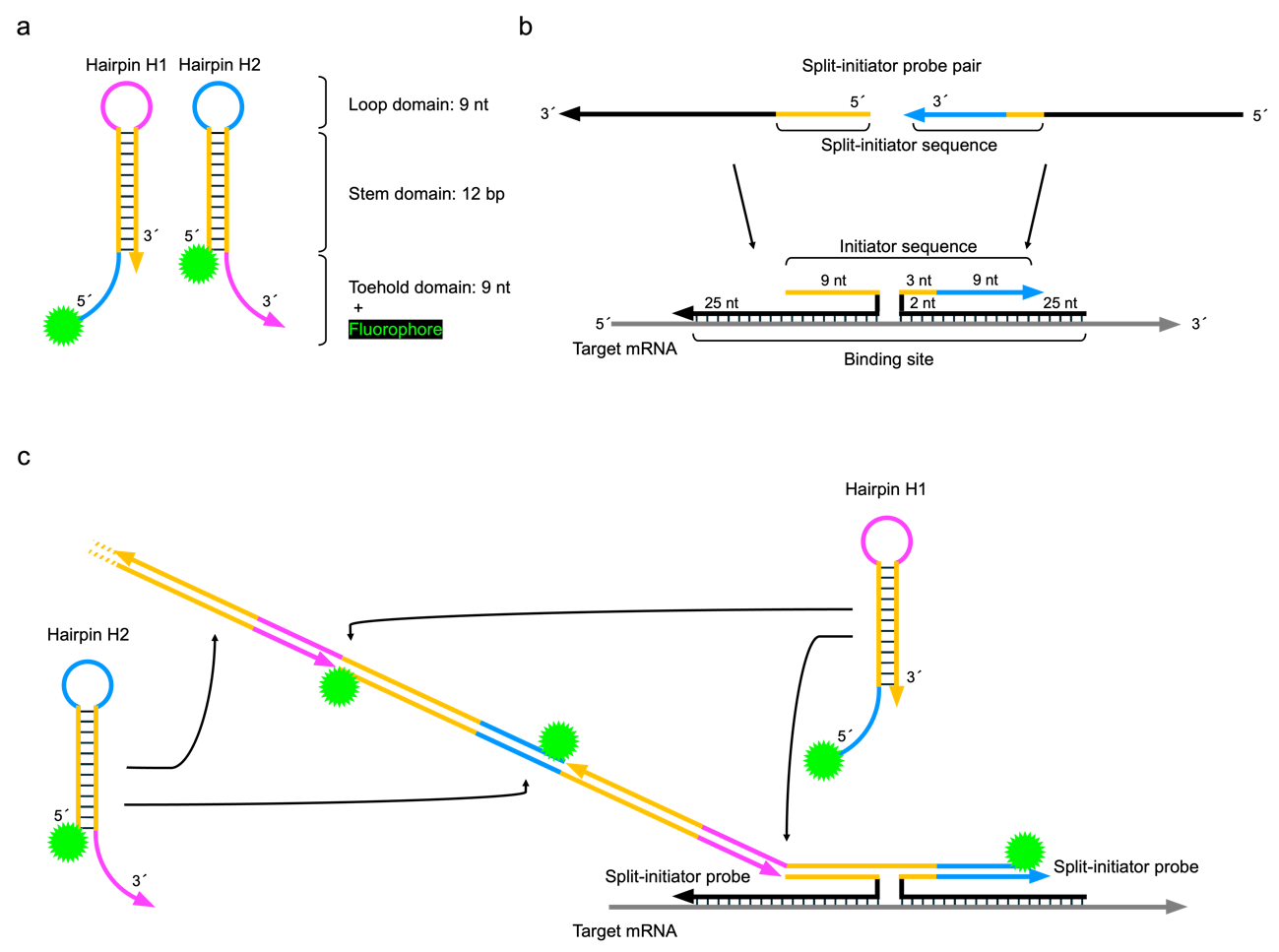


**Supplementary Figure S1. Schematic diagram of the is-shHCR.**

(a) Structure of hairpin DNA H1 and H2 conjugated to fluorophores (green). Arrowheads indicate the 3′ end of each strand. The toehold and loop domains are complementary between H1 and H2. bp: base pairs. nt: nucleotides. (b) Split-initiator-probe set. Each probe has a split-initiator sequence [9 nucleotides (nt) or 12 nt], a linker sequence (2 nt), and a sequence complementary to the target mRNA (25 nt). When the probes hybridize to the binding site of the target mRNA, they form an initiator sequence. (c) Hairpin DNA amplification using the hybridization chain reaction. When the toehold and stem domains of DNA H1 bind to the initiator sequence, DNA H1 and H2 are polymerized through a hybridization chain reaction.

**Supplementary Figure S2. Comparable signals were obtained using EC-isHCR with and without PBSTr rinsing and probe denaturation.**

Stage-16 embryos were stained with (a) and without (b) PBSTr rinsing before hybridization and denaturation of probes. Green signals indicate *piwi* mRNA. Scale bar: 100 µm.
